# Supplementary material for: Associations between Isolation Source, Clonal Composition, and Antibiotic Resistance Genes in Escherichia coli Collected in Washington State, USA
Source: Antibiotics (Basel). 2024 Jan 20;13(1):103. doi: 10.3390/antibiotics13010103 (PMC10812632; doi:10.3390/antibiotics13010103)
Supplement: Supplementary file 1 [file antibiotics-13-00103-s001.zip › antibiotics-2814627-File S1.pdf]

File S1: Accession numbers of sequences used in this analysis.
SRR10001251, SRR10451708, SRR10451709, SRR10451710, SRR10451711, SRR10451712, SRR10451715, SRR10451717, SRR10451718, SRR10451719, SRR10451720, SRR10451721, SRR10451725, SRR10503794, SRR10536656, SRR10536657, SRR10536658, SRR10536659, SRR10536660, SRR10536661, SRR10536662, SRR10536663, SRR10536664, SRR10536665, SRR10536666, SRR10536667, SRR10536668, SRR10536669, SRR10536670, SRR10536671, SRR10536672, SRR10536673, SRR10536674, SRR10537325, SRR10537328, SRR10537329, SRR10546708, SRR10546709, SRR10559557, SRR10559569, SRR10600402, SRR10600403, SRR10600523, SRR10600524, SRR10600525, SRR10609374, SRR10609375, SRR10609380, SRR10609382, SRR10609383, SRR10609386, SRR10609387, SRR10609388, SRR10609391, SRR10609392, SRR10609393, SRR10613301, SRR10613302, SRR10810132, SRR10810135, SRR10810139, SRR10810353, SRR10810362, SRR10880598, SRR10946714, SRR10946715, SRR10946716, SRR10947503, SRR10947731, SRR10947777, SRR10947785, SRR10947787, SRR10948120, SRR10948144, SRR10948212, SRR10948215, SRR10948217, SRR10948273, SRR10948361, SRR10948364, SRR10948365, SRR10948370, SRR10948371, SRR10959938, SRR10959939, SRR11029655, SRR11029832, SRR11029841, SRR11039029, SRR11039030, SRR11059186, SRR11059188, SRR11059190, SRR11059192, SRR11059194, SRR11059196, SRR11067917, SRR11067918, SRR11067919, SRR11068074, SRR11068075, SRR11068076, SRR11068077, SRR11068078, SRR11068079, SRR11068080, SRR11068084, SRR11068085, SRR11068086, SRR11068087, SRR11068088, SRR11068093, SRR11068159, SRR11091346, SRR11091347, SRR11091348, SRR11091349, SRR11091350, SRR11091351, SRR11091352, SRR11091353, SRR11091354, SRR11091355, SRR11091356, SRR11091357, SRR11091358, SRR11091359, SRR11091361, SRR11091362, SRR11091364, SRR11113559, SRR11113562, SRR11113564, SRR11113565, SRR11113566, SRR11113567, SRR11113568, SRR11113569, SRR11113570, SRR11113571, SRR11113572, SRR11113573, SRR11113574, SRR11114574, SRR11114575, SRR11114576, SRR11114577, SRR11114578, SRR11114579, SRR11114581, SRR11114627, SRR11114628, SRR11114629, SRR11116072, SRR11116073, SRR11116074, SRR11116075, SRR11116076, SRR11116083, SRR11116084, SRR11116085, SRR11116086, SRR11116087, SRR11116088, SRR11116089, SRR11116090, SRR11116092, SRR11116093, SRR11116094, SRR11116095, SRR11116096, SRR11116097, SRR11116098, SRR11116099, SRR11116100, SRR111161
