# Supplementary material for: Associations between Isolation Source, Clonal Composition, and Antibiotic Resistance Genes in Escherichia coli Collected in Washington State, USA
Source: Antibiotics (Basel). 2024 Jan 20;13(1):103. doi: 10.3390/antibiotics13010103 (PMC10812632; doi:10.3390/antibiotics13010103)
Supplement: Supplementary file 1 [file antibiotics-13-00103-s001.zip › antibiotics-2814627-supplementary.pdf]

(a)

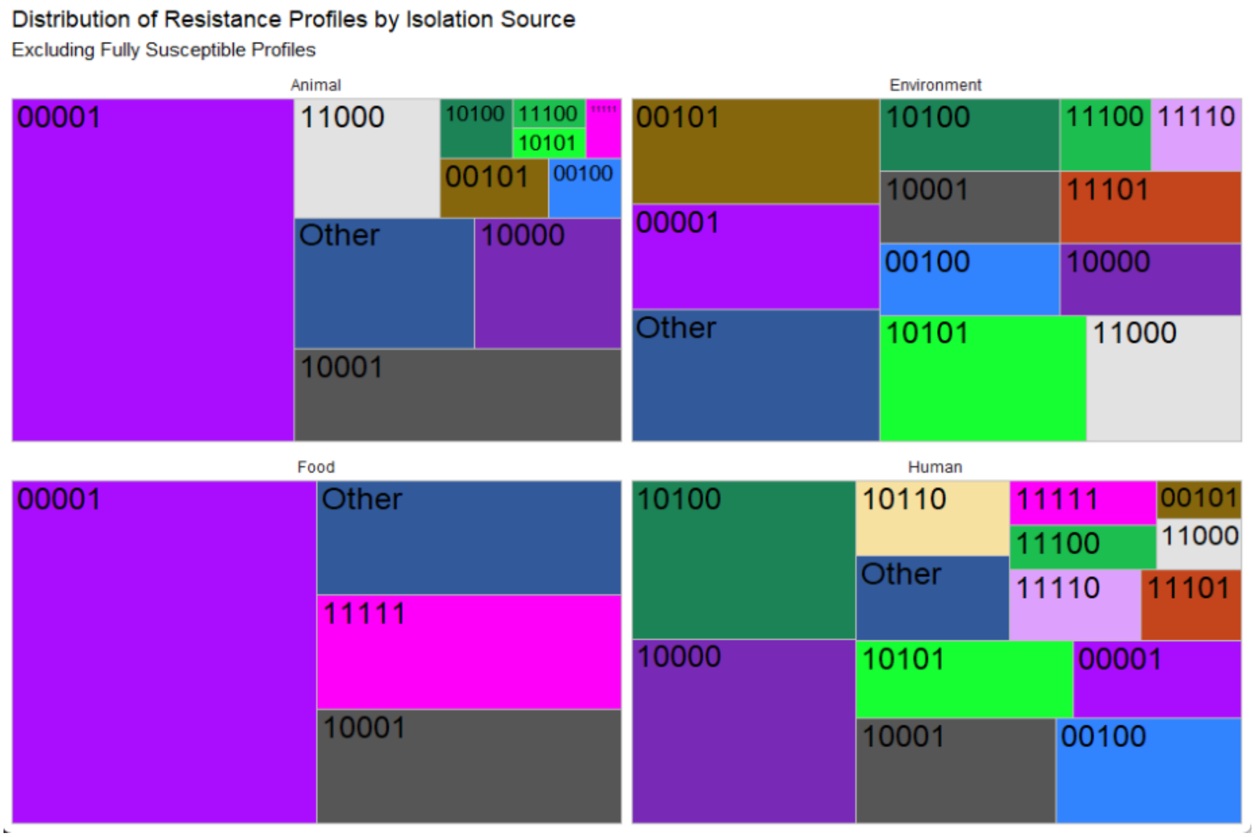

(b)

00000: No predicted resistance to any of the following: ampicillin, ceftazidime, ciprofloxacin, gentamicin, or tetracycline.
10000: Predicted resistance to ampicillin only.
01000: Predicted resistance to ceftazidime only.
00100: Predicted resistance to ciprofloxacin only.
00010: Predicted resistance to gentamicin only.
00001: Predicted resistance to tetracycline only.
11000: Predicted resistance to ampicillin and ceftazidime.
10100: Predicted resistance to ampicillin and ciprofloxacin.
10010: Predicted resistance to ampicillin and gentamicin.
10001: Predicted resistance to ampicillin and tetracycline.
01100: Predicted resistance to ceftazidime and ciprofloxacin.
01010: Predicted resistance to ceftazidime and gentamicin.
01001: Predicted resistance to ceftazidime and tetracycline.
00110: Predicted resistance to ciprofloxacin and gentamicin.
00101: Predicted resistance to ciprofloxacin and tetracycline.
00011: Predicted resistance to gentamicin and tetracycline.
11100: Predicted resistance to ampicillin, ceftazidime, and ciprofloxacin.
11010: Predicted resistance to ampicillin, ceftazidime, and gentamicin.
11001: Predicted resistance to ampicillin, ceftazidime, and tetracycline.

10110: Predicted resistance to ampicillin, ciprofloxacin, and gentamicin.
10101: Predicted resistance to ampicillin, ciprofloxacin, and tetracycline.
10011: Predicted resistance to ampicillin, gentamicin, and tetracycline.
01110: Predicted resistance to ceftazidime, ciprofloxacin, and gentamicin.
01101: Predicted resistance to ceftazidime, ciprofloxacin, and tetracycline.
01011: Predicted resistance to ceftazidime, gentamicin, and tetracycline.
00111: Predicted resistance to ciprofloxacin, gentamicin, and tetracycline.
11110: Predicted resistance to ampicillin, ceftazidime, ciprofloxacin, and gentamicin.
11101: Predicted resistance to ampicillin, ceftazidime, ciprofloxacin, and tetracycline.
11011: Predicted resistance to ampicillin, ceftazidime, gentamicin, and tetracycline.
10111: Predicted resistance to ampicillin, ciprofloxacin, gentamicin, and tetracycline.
01111: Predicted resistance to ceftazidime, ciprofloxacin, gentamicin, and tetracycline.
11111: Predicted resistance to all five of the following antibiotics: ampicillin, ceftazidime, ciprofloxacin, gentamicin, or tetracycline..

Figure S1: (a) Distribution of resistance profiles by isolation source, excluding fully susceptible profiles (profile 00000). (b) Definitions of resistance profiles in terms of predicted resistance.

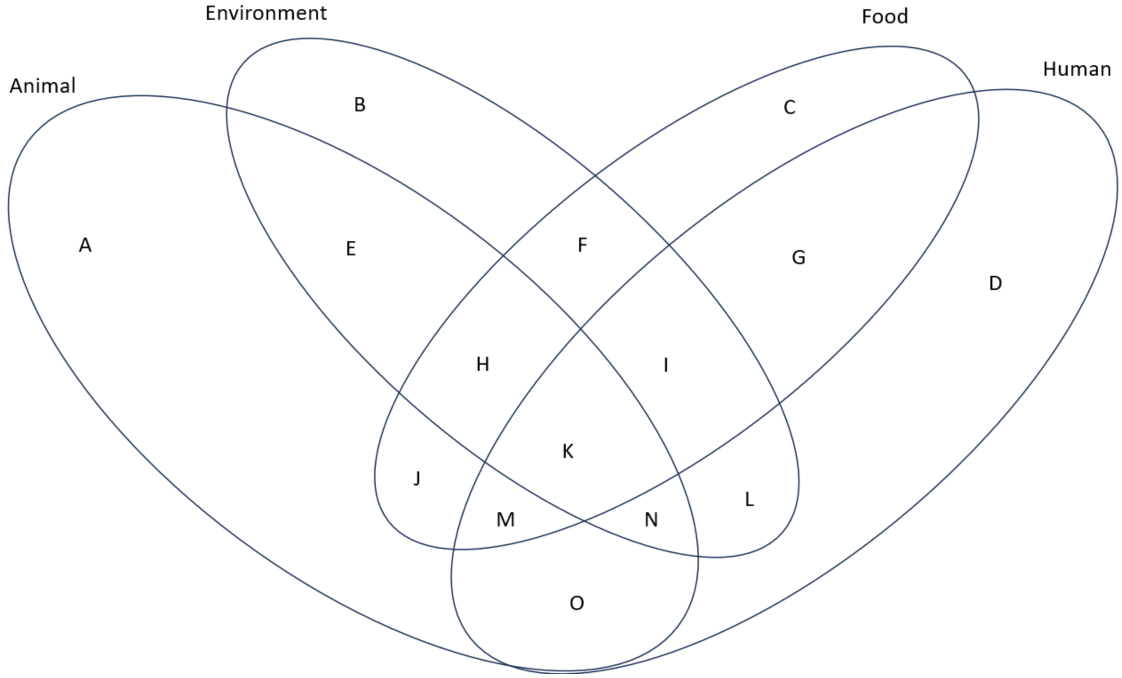

A. **Animal**: *aadA10*, aadA24, *aadA7*, *bla*$_{\textit{CTX-M-1}}$

B. **Environment**: *bla*$_{\text{DHA-1}}$, *bla*$_{\text{SHV-2a}}$, *lnu*(F), *qepA4*, *qnr*B4, *qnr*B67

C. **Food**: *aad*A22, *bla*$_{\text{NDM-5}}$, *bla*$_{\text{TEM-1D}}$, fosB1

D. **Human**: *aac(3)-IId*, *aac(6)-Ib-cr*, *aac(6)-Ib11*, *aad*A16, *aph(3)-IIa, aph(6)-Ic*, ARR-3, *bla*$_{\text{CARB-2}}$, *bla*$_{\text{CTX-M-14}}$, *bla*$_{\text{CTX-M-32}}$, *bla*$_{\text{CTX-M-55}}$, *bla*$_{\text{KPC-2}}$, *bla*$_{\text{OXA-1}}$, *bla*$_{\text{OXA-2}}$, *bla*$_{\text{OXA-9}}$, *bla*$_{\text{TEM-102}}$, *bla*$_{\text{TEM-182}}$, *bla*$_{\text{TEM-6}}$, *dfrA8, dfrA10, dfrA14, dfrA16, dfrA19*, dfrA27, *dfrA36, ere*(A), *erm*(B), *fosA, OqxA, OqxB*

E. **Animal and Environment**: *aac(2)-IIa*, *aac(3)-IV*, *aph(4)-Ia*, *bla*$_{\text{SHV-12}}$

F. **Environment and Food**: None

G. **Food and Human**: *tet*(D)

H. **Animal, Environment, and Food**: *floR*, *fosA7*

I. **Environment, Food, and Human**: None

J. **Food and Animal**: *catB3*

K. **Animal, Environment, Food, and Human**: *aac(3)-IIa*, *aac(3)*-*Via*, *aad*A2, *aad*A5, *aph(3)-Ia*, aph(3)-Ib, *aph(6)-Id*, *dfrA12*, dfrA17, *mph*(A), *sul1*, *sul2*, *tet*(A), *tet*(B)

L. **Environment and Human**: *bla*$_{\text{CTX-M-15}}$, *cmlA1*, *qnrS1*, *sul3*

M. **Animal, Food, and Human**: *bla*$_{\text{TEM-1A,}}$ *dfrA1*

N. **Animal, Environment, and Human**: *aadA1, bla*$_{\text{CMY-2}}$, *bla*$_{\text{TEM-1B}}$, *bla*$_{\textit{TEM}\text{-1C}}$, *catA1, dfrA5, qnrB19*

O. **Animal and Human**: *ant(2)-Ia*, *bla*$_{\text{CTX-M-27}}$, *dfrA7, tet*(C)

Figure S2 Venn diagram displaying the antibiotic resistance genes detected in isolates from animal, environment, food, and human sources.
